# A One-Step Extraction and Luminescence Assay for Quantifying Glucose and ATP Levels in Cultured HepG2 Cells

**DOI:** 10.3390/ijms19092670

**Published:** 2018-09-08

**Authors:** Rita Csepregi, Viktória Temesfői, Nikolett Sali, Miklós Poór, Paul W. Needs, Paul A. Kroon, Tamás Kőszegi

**Affiliations:** 1 Department of Laboratory Medicine, Medical School, University of Pécs, Ifjúság u. 13, H-7624 Pécs, Hungary; ritacsepregi93@gmail.com (R.C.); vtemesfoi@gmail.com (V.T.); niki26@gmail.hu (N.S.); 2 János Szentágothai Research Center, Ifjúság u. 20, H-7624 Pécs, Hungary; poor.miklos@pte.hu; 3 Department of Pharmacology, Faculty of Pharmacy, University of Pécs, Szigeti u. 12, H-7624 Pécs, Hungary; 4 Quadram Institute Bioscience, Norwich Research Park, Norwich NR4 7UA, UK; paul.needs@quadram.ac.uk (P.W.N.); paul.kroon@quadram.ac.uk (P.A.K.)

**Keywords:** Intracellular glucose assay, luminescence, validation, ATP, metabolic inhibitors, flow cytometry

## Abstract

A fluorescence-based enzymatic microplate intracellular glucose assay was designed and fully validated. The method was tested in a hepatocellular cancer cell line (HepG2). Our novel one-step extraction reagent gave stable cell lysates for glucose, adenosine triphosphate (ATP), and total protein determination from the same sample. Limit of detection for glucose was 0.13 µM (26 pmol/well), which is superior to commercially available glucose assays. Both intra- and interday assay imprecision in HepG2 cultures were less than 12% coefficient of variance (CV). In cell lysates spiked with glucose, recovery at two levels varied between 83.70% and 91.81%, and both linearity and stability were acceptable. HepG2 cells treated with agents affecting glucose uptake/metabolism (phloretin, quercetin, quercetin-3′-sulfate, NaF, 3-bromopyruvate, NaN_3_, oligomycin A, ochratoxin A, cytochalasin B, and anti-GLUT1 antibody) showed dose-dependent changes in glucose and ATP levels without total protein (cell) loss. Finally, we performed flow cytometric glucose uptake measurement in the treated cells using 2-[N-(7-nitrobenz-2-oxa-1,3-diazol-4-yl)amino]-2-deoxyglucose fluorescent glucose analog. Glucose uptake did not always mirror the intracellular glucose levels, which most likely reflects the differences between the two methodologies. However, interpreting data obtained by both methods and taking ATP/protein levels at the same time, one can get information on the mode of action of the compounds.

## 1. Introduction

Glucose metabolism plays a crucial role in the viability of all cell types. Currently, glucose-based viability assays are quite infrequent. This may be due to the phenomenon that cultured cells consume glucose from the medium quite rapidly, therefore culture conditions are often difficult to standardize, allowing comparison between different cell treatments. However, some analytical approaches in the literature can be found for detecting and quantifying intracellular glucose using nonenzymatic techniques such as chromatographic and electrophoretic based systems (HPLC and GC with detection limit from 10^−4^ M to 1 mM glucose) and coupled enzymatic systems such as glucose oxidase/peroxidase and hexokinase/glucose-6P-dehydrogenase, which are glucose-specific and easier to perform than nonenzymatic approaches. Glucose oxidase (GOD) assays produce H_2_O_2_, therefore in this technique peroxidase (POD) is added to drive the oxidation of a chromophore. The kits contain a wide variety of fluorescence or visible light chromophores, such as Amplex Red (Em: 585 nm), phenol/4-aminoantipyrine (A: 514 nm), and o-dianisidine (A: 540 nm). The GOD–POD coupled reaction has good precision over a dynamic range of 1–20 mM glucose, but increasing the sensitivity of detection it can reach 3 µM [1,2,3]. In the field of nanochemistry, an alternative glucose-sensing fluorophore was synthesized, carbon nanodots, which are able to quantify micromolar glucose concentrations [4]. In a novel colorimetric method using GOD coupled with peroxidase-like activity of ficin as a catalyzer and the peroxidase substrate 3,3′,5,5′-tetramethylbenzidine (TMB) in the presence of H_2_O_2_, glucose detection over a range of 2–100 μM was possible with a detection limit of 0.5 μM [5]. Moreover, live imaging of intracellular glucose levels and/or uptake has also been demonstrated recently [6].

Inevitably, liver cells play a major role in glucose metabolism and regulation. Therefore, cultures of cells of liver origin, such as HepG2 cancer cells, are good candidates for testing glucose metabolism and uptake under experimental conditions [7]. Cancer cells have the ability to change the pathway of energy production, allowing them to utilize glycolysis alongside oxidative phosphorylation to sustain their high rates of proliferation even in anaerobic conditions, based on the Warburg effect [8]. Additionally, the overexpression of glucose transporters in cancer cells results in increased glucose uptake. Classical studies for the assessment of glucose uptake used radioactive glucose analogs [9]. More recently, fluorescent glucose analog 2-[N-(7-nitrobenz-2-oxa-1,3-diazol-4-yl)amino]-2-deoxyglucose (2-NBDG) and 6-NBDG were introduced to measure glucose uptake rates in normal and tumor cell lines in vitro. 2-NBDG was shown to be transported to the cytoplasm by the same glucose transporters (GLUTs) as glucose, and the kinases (hexokinase) are able to phosphorylate it at a certain rate as “normal” glucose, which leads to its degradation into a nonfluorescent derivative. It was also reported that the fluorescence intensity detected from the cells represents a dynamic equilibrium of uptake and decomposition of the compound [7,10,11,12,13].

Adenosine triphosphate (ATP) is a functional dynamic endpoint of mitochondrial respiration. Intracellular ATP contents are sensitive indicators for the assessment of toxicological effects when testing metabolic inhibitors [14]. Despite the fact that ATP levels are quite constant in living cells, intracellular ATP can degrade very rapidly after cellular injury. Therefore, when measuring cellular ATP levels, it is of utmost importance to use a suitable extraction medium that stabilizes the released ATP from the lysed/fixed cells. For appropriate evaluation of ATP levels, results should be referred to a cellular parameter with much more stability than ATP. This might be total cell protein and/or nucleic acid content [15,16].

Because in most cases the measured viability parameters are intracellular, they must be released from the cells without loss or degradation. There are several types of cell disruption techniques, including physical and nonphysical methods. The physical systems require specific conditions, including high pressure, temperature, and energy. However, nonphysical methods such as detergents, solvents, or enzymes are more widely available in laboratories and directly damage the cell membrane, leading to the release of intracellular components [17]. One of the most commonly used cell lysis detergents in toxicological/viability studies is Triton X 100, a nonionic surfactant that is able to permeabilize the membrane, extracting the intracellular (noncytoskeletal) proteins and partially stabilizing the intracellular components [18]. When using lytic agents, it is of utmost importance to extract the cells as rapidly as possible while stabilizing the intracellular components intended to be analyzed.

In the present research, our main goal was to establish and validate a fluorescence intracellular microplate glucose determination method of high sensitivity with simultaneous ATP and total cellular protein quantification. We developed a microplate-based glucose assay with a one-step detergent extraction using HepG2 cell cultures treated with different glucose uptake/metabolism inhibitors as the test system. Further, we show that by measuring uptake of the glucose analog 2-NBDG by flow cytometry in parallel with the microplate assay for cellular glucose, ATP, and total protein levels, information on the mode of action of different test compounds that might influence glucose homeostasis can be obtained.

## 2. Results

### 2.1. Validation Data

**Figure 1:**
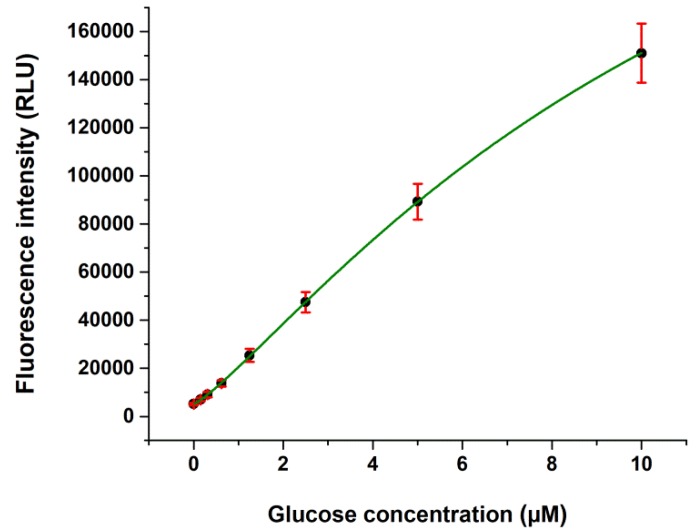
Example cumulative calibration curve for the glucose assay. Mean ± standard deviation (SD) data from 88 separate calibrations in the range of 0.15–10 µM glucose are shown. With logistic model fitting, the equation of the regression line is: *y* = 340441.8 + (5180.88-340441.8)/(1+ (*x*/12.4)^1.21^, *R*^2^ = 0.9997.

The cumulative glucose calibration curve obtained from 88 independent calibrations is shown in Figure 1. The calibrated working range was between 0.15 and 10 µM. We used a logistic nonlinear curve fitting for the calibrator data. Limit of blank (LOB), limit of detection (LOD), and limit of quantification (LOQ) of our glucose assay were 0.078, 0.130, and 0.362 µM, respectively.

**Figure 2:**
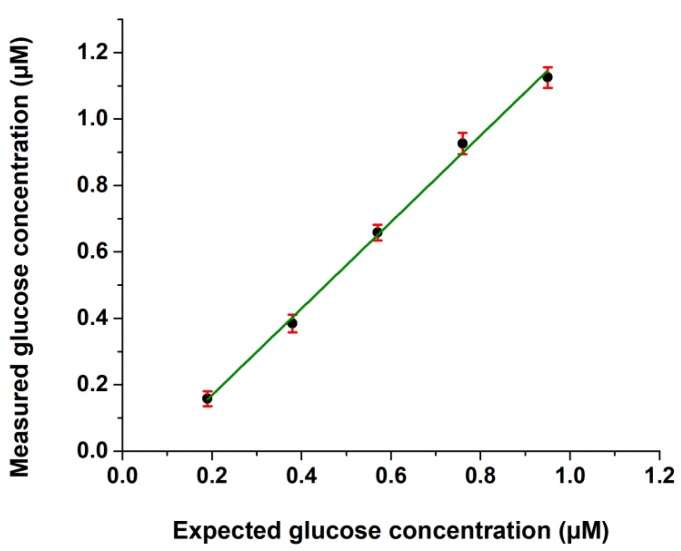
Linearity of the glucose assay. Linearity was calculated by diluting cell extracts within the range of 20–100% of their original glucose concentrations using five different dilutions. The linear regression equation is: *y* = 1.2381*x* − 0.0925, *R*^2^ = 0.9974. Mean values of three parallel measurements (eight technical replicates for each dilution) are demonstrated.

**Figure 3:**
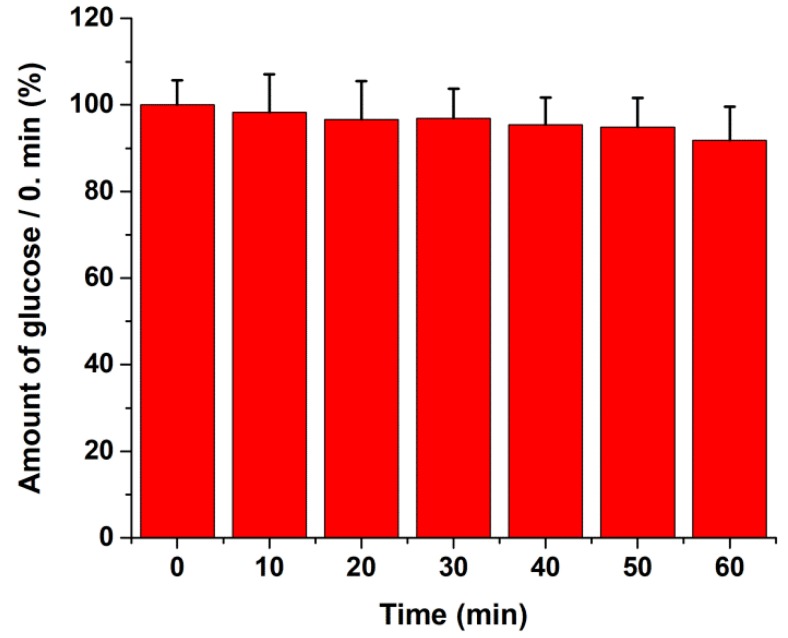
Stability study of glucose in the intracellular glucose assay using the glucose extraction (GEX) solution. HepG2 cells, mean values ± SD of eight technical replicates at each time point are shown.

Both intra- and interassay imprecision in the aqueous environment was less than 10%, expressed as CV, and recovery of the glucose assay was found to vary between 89.80% and 102.09%, as shown in Table 1. For the untreated cells, we detected approximately 1 µM intracellular glucose concentration in the wells, and the recovery of our intracellular glucose assay was found to vary between 83.70% and 91.81% at two concentration levels (Table 2). The assay showed good linearity (*R*^2^ = 0.997) in the range of 0.2–1 µM glucose in the cell extracts (Figure 2). During 60 min storage of HepG2 lysates in glucose extraction (GEX) solution, we did not find a significant loss of glucose (<5%) (Figure 3).

### 2.2. Intracellular Glucose, ATP, and Protein Measurements

**Figure 4:**
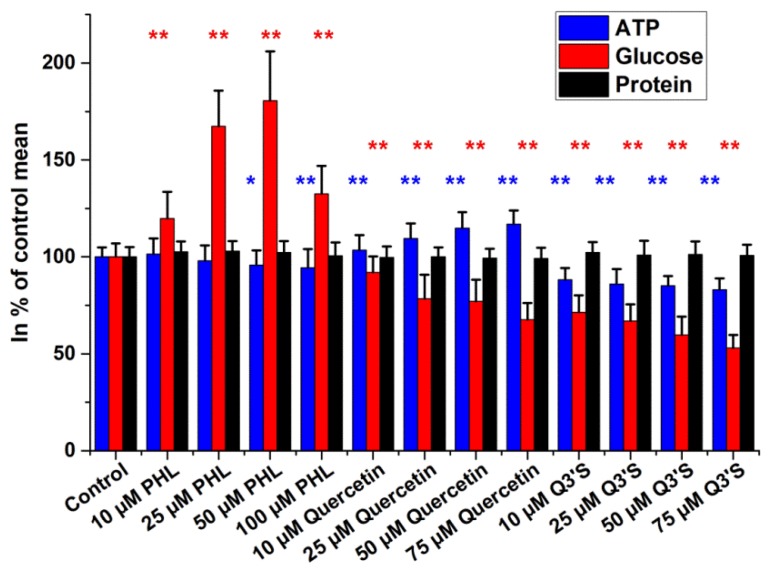
Intracellular glucose, ATP, and total protein levels of HepG2 cells after 4 h treatment with inhibitors of glucose transporters (GLUTs). Data are expressed as % of control. Bars represent mean ± SD of six independent experiments (* *p* < 0.05, ** *p* < 0.01 compared with controls).

To test the efficiency of the modified glucose assay, cells were treated with different inhibitors of glucose uptake/metabolism for four hours in Dulbecco’s Modified Eagle’s Medium (DMEM). The effects of inhibitors of glucose transporters were investigated by using three polyphenol compounds (quercetin, quercetin-3′-sulphate (Q3′S), and phloretin), as shown in Figure 4. Total cellular protein contents remained unchanged in response to all three treatments. The most striking alteration was observed after phloretin exposure, where intracellular glucose increased rapidly in a dose-dependent manner. It is noteworthy that 50 µM phloretin elevated glucose levels to approximately 180% of those of the controls. Quercetin and its major human metabolite, Q3′S, normally found in human blood plasma, caused a decrease in glucose content of HepG2 cells, with the metabolite being the most active. ATP levels decreased slightly at 50 µM and 100 µM concentrations of phloretin; however, Q3′S caused more substantial ATP depletion, while quercetin caused an increase in intracellular ATP levels.

**Figure 5:**
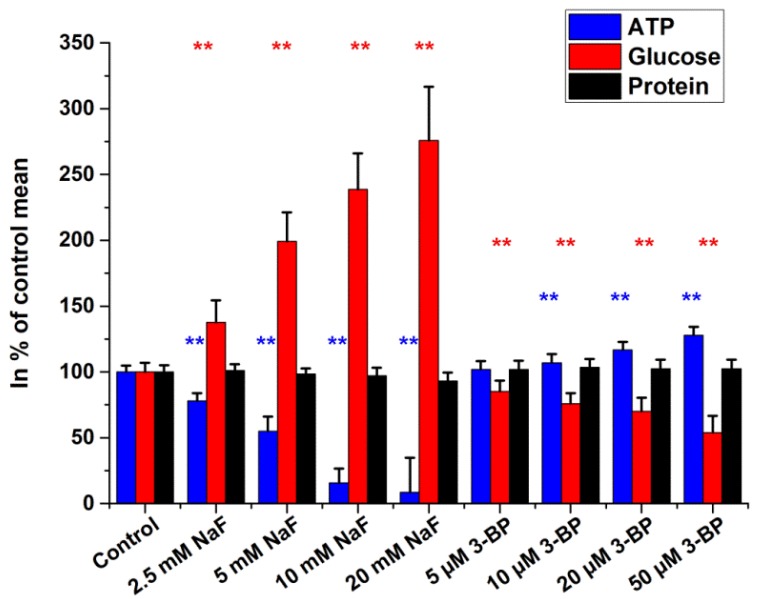
Glucose, ATP, and protein levels of HepG2 cells treated with glycolysis inhibitors. Cells were incubated for 4 h. Data are expressed as % of control. Bars represent mean ± SD of six independent experiments (* *p* < 0.05, ** *p* < 0.01 compared with controls).

Figure 5 illustrates the effects of glycolysis inhibitors (NaF and 3-bromopyruvate). NaF exerted marked ATP depletion in a dose-dependent manner. On the other hand, intracellular glucose levels showed the accumulation of unmetabolized glucose in the samples. At the highest concentration of NaF (20 mM), ATP level was approximately 9% and glucose was 275% of the control value. Opposite to NaF, 3-bromopyruvate (3-BP) increased ATP contents slightly while decreasing intracellular glucose. No change in protein concentration was detected.

**Figure 6:**
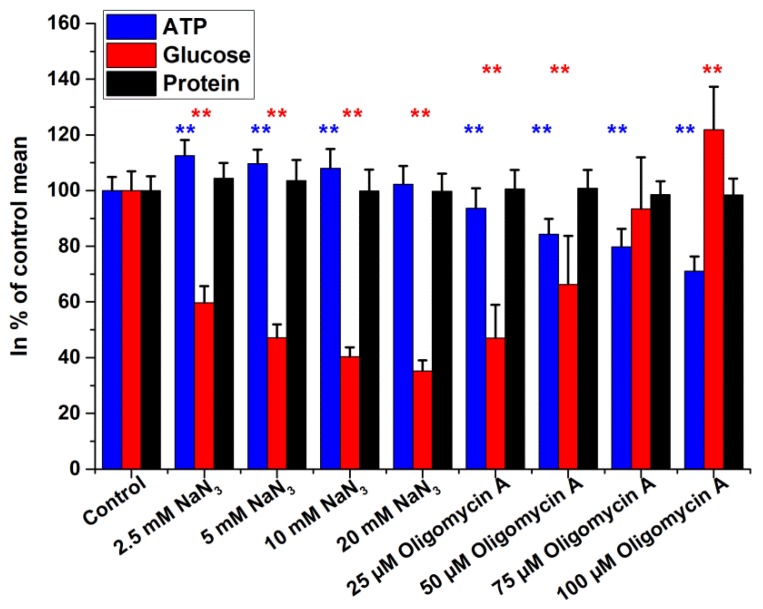
Intracellular glucose, ATP, and protein levels of HepG2 cells after 4 h treatment with inhibitors of terminal oxidation. Data are expressed as % of control. Bars represent mean ± SD of six independent experiments (* *p* < 0.05, ** *p* < 0.01 compared with controls).

Effects of NaN_3_ and oligomycin A treatments are presented in Figure 6. Glucose contents decreased after NaN_3_ and increased after oligomycin A exposure. For NaN_3_, we observed a dose-dependent increase of ATP, while oligomycin A caused dose-dependent ATP depletion. There was no change in total cellular protein contents in the treated samples.

**Figure 7:**
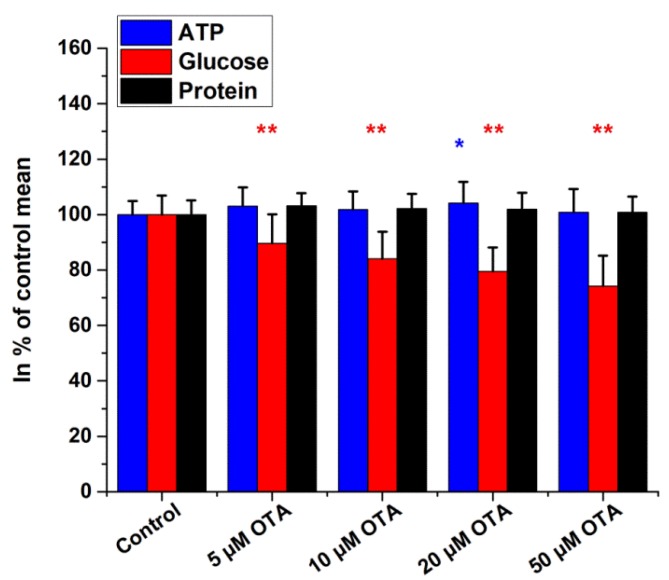
Intracellular glucose, ATP, and protein contents of HepG2 cells treated with ochratoxin A (4 h incubation). Data are expressed as % of control. Bars represent mean ± SD of six independent experiments (* *p* < 0.05, ** *p* < 0.01 compared with controls).

Data obtained for ochratoxin A (OTA) exposure are demonstrated in Figure 7. We observed a slight dose-dependent decrease in ATP contents, while glucose and protein levels remained unchanged.

**Figure 8:**
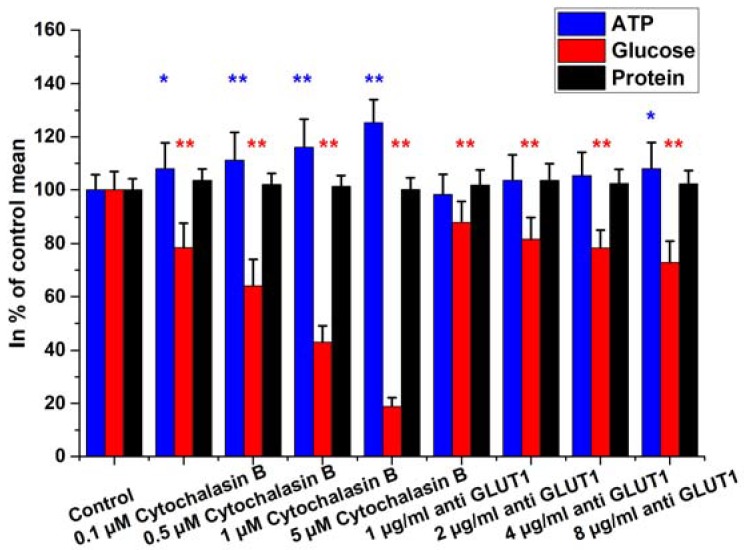
Intracellular glucose, ATP, and protein contents of HepG2 cells treated with cytochalasin B and anti-GLUT1 antibody (4 h incubation). Data are expressed as % of control. Bars represent mean ± SD of six independent experiments (* *p* < 0.05, ** *p* < 0.01 compared with controls).

Finally, the GLUT proteins were inhibited with anti-GLUT1 antibody and cytochalasin B. Anti-GLUT1 treatment within the concentration range of 1–8 µg/mL caused a dose-dependent response in the glucose content of the HepG2 cells. The effect of cytochalasin B was more pronounced than that of the specific antibody and was strongly concentration-dependent (0.1 µM–5 µM; Figure 8).

### 2.3. Extracellular Lactate Levels

The growth of untreated cells increased lactate levels in the medium approximately twofold compared with medium alone (no cells), as shown in Table 3. Treatment with phloretin, quercetin, and Q3′S caused minor changes only in the lactate levels of the medium, whereas glycolysis inhibitors (NaF, 3-BP) induced lactate depletion. Notably, the highest concentration of NaF tested (20 mM) reduced lactate concentration to approximately 30% of the control value. The general inhibitors of terminal oxidation did not affect lactate production in a uniform manner; for example, NaN_3_ increased lactate, whereas oligomycin A did not change lactate concentrations. Similar to oligomycin A, OTA caused no change in lactate levels. Lactate was not estimated after antibody and cytochalasin B treatments.

### 2.4. Glucose Uptake

**Figure 9:**
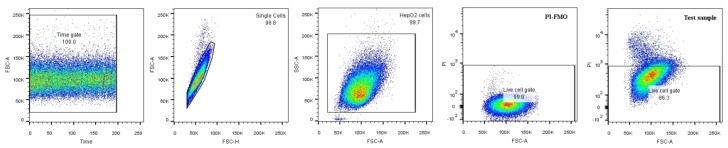
Gating strategy in the flow cytometry analysis.

The fluorescent glucose analog uptake assay was performed in triplicate. The propidium iodide (PI) negative and positive populations were defined based on fluorescence minus one (FMO) controls to include the contribution of one fluorophore to the detection of another during the measurement. We did not differentiate between apoptosis and necrosis. Using PI, we could estimate the percentage of late apoptotic and necrotic cells in the samples. In the statistical analysis, we excluded cells that showed PI positivity and used the PI negative population (Figure 9).

**Figure 10:**
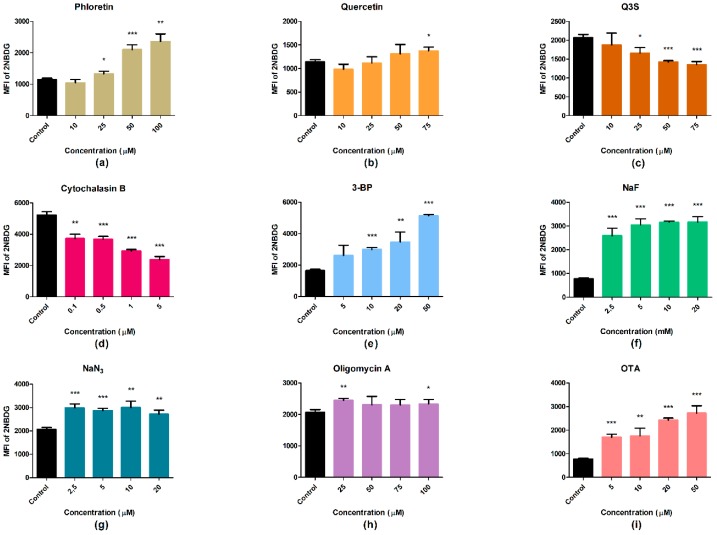
2-[N-(7-nitrobenz-2-oxa-1,3-diazol-4-yl)amino]-2-deoxyglucose (2-NBDG) uptake in HepG2 cells as a result of (**a**) phloretin, (**b**) quercetin, (**c**) Q3′S, (**d**) cytochalasin B, (**e**) 3-BP, (**f**) NaF, (**g**) NaN_3_, (**h**) oligomycin A, and (**i**) OTA treatment. Incubation time with metabolic inhibitors: 4 h, 2-NBDG: 1 h. MFI, mean fluorescence intensity. Columns represent the mean of medians, error bars show the interquartile range of fluorescence intensity of intracellular 2-NBDG. PI-positive cells are excluded from the analysis. (* *p* < 0.05, ** *p* < 0.01, *** *p* < 0.001 compared with controls.)

Although phloretin and quercetin are reported as glucose transporter inhibitors, we could observe a slight decrease in uptake only at the lowest concentrations following treatment with these polyphenols. A dose-dependent increase was observed from the 25 µM dose. The quercetin metabolite Q3′S decreased the uptake of the glucose analog in a dose-dependent manner similar to cytochalasin B, which proved to be the most effective. The glycolysis inhibitors 3-BP and NaF caused increases in uptake depending on their concentration. Oligomycin A and NaN_3_ elevated the uptake level significantly compared with the control, but the alteration was not dose-dependent. OTA, as a generally toxic agent, seemed to cause significantly increased glucose uptake (Figure 10).

Glucose uptake was also investigated without excluding the dead cell populations. In Supplementary Figure S1, a similar tendency to that for the live cells only can be observed. These data contain the passively diffusible 2-NBDG molecules together with the transported ones.

## 3. Discussion

In this study, our major aim was to work out a multiparametric viability test with a one-step extraction method that solubilizes cellular proteins with simultaneous release and stabilization of cellular glucose and ATP from HepG2 cells. The fluorescence-based enzymatic glucose assay was fully validated and adapted to microplates. Our intracellular glucose measurement protocol is based on the formation of H_2_O_2_ and subsequent oxidation of the stable resorufin derivative in the glucose reagent, as described by Zhou et al. in 1997 [19]. Our ATP determinations were made by using a commercial bioluminescence kit and an already published extraction method [15,20]. The major goal of the multiparametric approach is that during the extraction, both cellular ATP and glucose remained stable at least up to the incubation time required for the measurements (5 min for ATP and 30 min for glucose). Regarding the ATP assay itself, the most important step is extraction to give the maximum yield without deterioration. One problem might arise from the minimal matrix difference between the standards (without cell lysates) and the cellular samples (with cell lysates), because the bioluminescence reaction is sensitive to microenvironment. This problem might be overcome by a standard addition technique [15], which would make the microplate assay complicated and time-consuming.

Our method is superior to similar ones that are currently commercially available. Comparing the LOD of our technique with that of several other methods, there is a wide choice in both principles and sensitivities. The nonenzymatic copper-iodometric test shows a 0.05 µM glucose detection limit [3], while chromatographic systems (HPLC, GC) depend on the type of detector as well. Different LODs are as follows: refractive index 0.1–300 µM [21], pulsed amperometry 0.2 µM [22], evaporative light scattering 0.002 µM [23], and UV/fluorescence 0.05 µM [3]. Certain nonenzymatic tests have better sensitivity than ours, but it is important to keep in mind that these techniques are suitable for detecting glucose concentration in serum, blood, or urine only. In this regard, the enzymatic approaches are more versatile, because in addition to blood and urine, they are able to detect glucose in complex cellular systems as well. In spite of the need to determine intracellular glucose, there is no gold standard technique for it. A major problem comes first with the extraction that should stabilize glucose levels at least during the assay time. In particular, GOD/POD reactions are the most popular techniques in the commercial kits [3]. The sensitivity of these kits usually covers a dynamic range of 1–20 mM glucose [1], but depending on the detection method, an LOD of 3 to 5.2 µM can be achieved [2,4]. Amplex Red enzymatic fluorimetric assay is the most common commercially available method from different manufacturers, with an analytical sensitivity at about 1 µM [24,25]. Up to now, the most sensitive commercial kit has been able to detect glucose with an LOD of 0.5 µM [26]. Our proposed technique has an LOD of 0.13 µM glucose, which is almost 5 to 10 times more sensitive than most commercial assays. Furthermore, our extraction reagent stabilizes ATP as well, as demonstrated earlier [15,20]. In the validation process, we studied the analytical performance of our method by using in-house glucose standards and a commercially available calibrator for automated systems (Cfas Roche, Mannheim) and serum-based quality control (PreciControl Universal, Roche, Mannheim) with target values given for the hexokinase/glucose-6P-dehydrogenase method. In our study, both the homemade and commercially available calibrators/controls gave identical imprecision and recovery data.

The present assay detects 26 pmol/well glucose as the limit of detection. This LOD has been overcome in special fluorescence single-cell glucose monitoring techniques [27,28]; however, this approach needs special fluorescence microscopy imaging instrumentation and/or fluorescence correlation spectroscopy with a low-throughput facility.

To test the discriminating ability of our assay, we applied treatments that are known to affect cellular metabolic pathways at several points regarding glucose uptake and utilization. The proposed assay sensitively detected the effects of various compounds. Additionally, we used the fluorescent glucose analog 2-NBDG to investigate glucose uptake by flow cytometry. Analysis of the results was carried out both for the live cells only (PI-negative populations) and for all cells (without excluding the PI positive population). The two sets of data showed a similar tendency, indicating negligible interference of the studied cells with compromised membranes. The ungated samples are considered to be similar to the microplate assays where there is no possibility to selectively analyze the living cells only. All the compounds we applied are reported as glucose uptake and/or metabolic inhibitors that can cause cell death depending on treatment time and concentration [29,30].

As the first line, the glucose transporter inhibitors phloretin, quercetin, quercetin metabolite Q3’S, and cytochalasin B were tested. NaF and 3-BP were applied as glycolysis inhibitors. We used NaN_3_ and oligomycin A as interfering agents with terminal oxidation and OTA as a generally toxic compound. We selectively inhibited the GLUT1 transporter protein with antibody treatment, but it is hypothesized that the antibodies could reach only the spatially available GLUT1 molecules on the surface of the cells.

In tumor cells, such as the HepG2 cell line, glycolysis produces most of the ATP even when oxygen supply is sufficient and the mitochondrial pathways are functioning. The Warburg effect leads to elevated lactate production [8,31,32]. Under these conditions, glucose is the main source of energy if available. Inhibition of nutrient transporters, especially the GLUTs and elements of the glycolysis pathway, causes general imbalance of cellular metabolism, which can lead to serious effects [30]. Inhibition of glucose uptake with polyphenols, quercetin, and Q3’S resulted in a change of intracellular glucose content and ATP production in the microplate assay. The cells utilized the available glucose molecules, and Q3’S decreased while quercetin slightly increased the ATP levels in a dose-dependent manner at the end of the treatments. The cells became starved, even if the medium was supplemented with other nutrients ready for use. One of the applied polyphenols, phloretin, did not seem to decrease the glucose content in the microplate assay. It is reported to be less effective than its glycoside regarding affinity toward glucose transporters [33].

We observed a dose-dependent increase of 2-NBDG uptake following quercetin treatment as well, although at the lowest concentration a decrease was observed. Q3′S and cytochalasin B were the only compounds that inhibited 2-NBDG uptake in HepG2 cells in all tested concentrations. It is quite interesting, because Q3′S is the main metabolite of orally consumed/administered quercetin in the human circulation [34]. Despite conjugated metabolites being commonly inactive, previous studies highlighted that Q3′S binds to albumin with similar affinity as the parent compound and also inhibits the CYP2C9 enzyme [35,36].

Most of the cancer cells increased their glucose transport and glycolysis compared with normal cells, which was revealed by positron emission tomography (PET) scan and other detection methods [37]. Our investigated GLUT inhibitors phloretin, quercetin, and its main metabolite Q3′S decreased glucose absorption through GLUT2, which is expressed in HepG2 cells. Cytochalasin B is considered to affect GLUT1–4 transporters [38], which was demonstrated by both the microplate and flow cytometry assays. Nevertheless, GLUT1 and GLUT3 expression is also upregulated in different types of cancers [39]. Therefore, the unexpected data in the case of phloretin might be explained by the nonuniform inhibition of all transporters in the sample, such as GLUT1, which can be inhibited by specific antibodies only. Some studies have proved that GLUT1 antibody treatment is effective in reducing tumor cell growth in vitro [40]. Our results of anti-GLUT1 treatment support the potential antitumor effect of glucose transporter inhibition. Although the reason for the different effects of these flavonoids is unclear, we postulate that this might be associated with macropinocytosis of glucose by cancer cells from the extracellular medium. Qian et al. recently reported that KRas^mut^ genotype is associated with a phenotype where macropinocytosis is facilitated, which causes nonspecific uptake of extracellular molecules such as glucose, amino acids, and lipids. The HepG2 cell line possesses mutated oncogenic Ras isoforms [41]. This assumption may explain the unexpected low or lack of effect of some polyphenols known as glucose uptake inhibitors. Thus, it is important to determine the genotype (such as KRas status) of the tumor cells, not only the phenotype of GLUTs upregulated in the cells under investigation.

NaF and 3-BP are inhibitors of the glycolysis pathway. The effect of NaF may differ depending on cell type, concentration, and duration of treatment. Fluoride in millimolar concentration affects mainly the glycolytic enzyme system by decreasing its activity [42]. NaF treatment elevated intracellular glucose levels and augmented uptake was observed, while the amount of ATP showed a significant decrease. The general inhibition of the enzymatic network involved in glucose metabolism caused the accumulation of glucose and 2-NBDG molecules that entered the cells. The rate of accumulation increased dose-dependently, in parallel with the concentration of NaF. Due to the enzymatic arrest, cellular ATP production, and thus viability, decreased as well [43,44]. 3-BP inhibits the hexokinase II enzyme, which catalyzes the first step of glucose utilization [30]. Following treatment, intracellular glucose showed lower levels, while the intensity of uptake and the amount of ATP were elevated. Comparing these two substances, in the case of NaF we observed a general inhibition of metabolic enzymes. It can be assumed that in the case of NaF, the utilization of glucose and other nutrients is prevented, which is supported by the decreased lactate level at an NaF concentration of 20 mM compared to 2.5 mM. During the inhibition of hexokinase II by 3-BP, other routes and enzymes could continue to function, which, taking into account the needs of the cell, may complement the lost pathway or enzyme activity. The cells were able to decompose their glucose content, therefore we could see a moderate increase in the ATP level and a decrease in lactate production compared with the control.

The terminal oxidation inhibitor oligomycin A elevated the uptake significantly, but not in a dose-dependent manner. We can see an elevation in intracellular glucose levels, which may come from the blocked utilization and inhibition of ATP synthase [45]. The ATP level decreased significantly as well compared with the control. NaN_3_ showed similar characteristics as oligomycin A in the flow cytometry experiments, but in the plate reader assay the intracellular glucose level decreased steeply in a dose-dependent manner. ATP content at the lowest concentration was significantly higher, but tended to decrease back to the level of the control.

As a response to the toxic effects of OTA [46] in HepG2 cells, we observed that lactate production, and thus glycolysis, at lower concentrations of the toxin were elevated, while the glucose content decreased. The cells utilized the intracellular sugar. At higher concentrations, glucose content was further reduced and lactate production did not change, while 2-NBDG uptake was increased depending on the regulatory mechanism [47].

When the available intracellular glucose starts to decrease, the cells (through sensing mechanisms) upregulate the expression of transporters and the expression and activity of enzymes and transcription factors required for the utilization of nutrients [48,49,50,51]. Apart from sensing glucose and glycolysis intermediates, the regulatory mechanisms can also be implemented by other processes. Control is carried out through positive and negative feedback and feed-forward mechanisms [48]. Exploring the intervention of the applied compounds in the intracellular metabolic networks and signaling pathways requires other associated studies. In this study, we used these compounds to test our assay that was developed and adapted to the microplate reader. The relationship between the glucose content of cells and the intensity of uptake was not always as expected. The two types of tests show that the intervention point and manner of action of the metabolic inhibitors depend largely on the concentration and duration of treatment. One should keep in mind that the microplate assay does not discriminate between live and dead cells, but is suitable for fast screening. In the flow cytometry experiments, we had to have an extra 1 h incubation with 2-NBDG without the treating agents present. This standard difference may also explain the observed alterations between the two types of assays. 2-NBDG is a larger molecule than the glucose or 2-deoxy glucose that is used in radioactive uptake measurements. Therefore, the two methods are not identical and are not directly comparable. Recently, a novel bioluminescence 2-deoxy glucose uptake assay was introduced for microplate readers, but this method is not suitable for flow cytometry [52,53,54].

The measured substantial changes in ATP levels with no apparent cell loss (no change in protein content) suggest that cellular viability assays should be multiparametric for more precise assessment of the effects of cytoprotective/cytotoxic compounds.

## 4. Materials and Methods

### 4.1. Chemicals

Phloretin, quercetin, 3-bromopyruvate, cytochalasin B, rabbit polyclonal GLUT1 antibody, oligomycin A, ochratoxin A, Dulbecco’s Modified Eagle’s Medium (DMEM), Ampliflu™ Red (10-acetyl-3,7-dihydroxyphenoxazine), propidium iodide (PI), trypsin-ethylenediaminetetraacetic acid (EDTA), penicillin–streptomycin for cell culture, glucose oxidase (GOD), and peroxidase (POD) were purchased from Sigma-Aldrich. 2-NBDG was from Invitrogen-Fisher Scientific. Bioluminescent ATP Assay Kit CLSII and peroxide-free Triton X 100 (TX-100) were from Roche. Sodium fluoride and sodium azide were from Acros Organics. To wash the cell cultures, magnesium and calcium containing phosphate-buffered saline (PBS, pH 7.4) was used. Our ATP buffer was made of 0.1 M Tris-base containing 2 mM EDTA-Na_2_ and 10 mM Mg_2_SO_4_ (Lach-Ner, Neratovice, Czech Republic), adjusted to pH 7.75 by glacial acetic acid. For total protein determination, homemade Bradford reagent [55] was applied. Fetal bovine serum (FBS, Pan-Biotech, Aidenbach, Germany) and bovine serum albumin (BSA, Biosera, Nuaille, France) were used as received. Quercetin-32032-sulfate (Q3′S) was synthetized based on earlier communication [56]. All sterile plasticware was from Greiner. For optical measurements, standard 96-well, white 96-well, and 6-well microplates were used (Sarstedt, Nümbrecht, Germany).

### 4.2. Cell Culture and Treatments

Experiments were carried out with HepG2 cells (ATCC: HB-8065), a hepatocellular carcinoma cell line derived from a human individual. The adherent cells were cultured in DMEM with high glucose (4500 mg/L) supplemented with 10% FBS, penicillin (100 U/mL), and streptomycin (100 µg/mL) in 75 cm^2^ cell culture flasks at 37 °C in a humidified atmosphere containing 5% CO_2_. Cells were then trypsinized and plated onto 96- or 6-well sterile plastic plates (10^4^ or 6 × 10^5^ cells/well, respectively). Experiments were performed on cells that were 80–90% confluent, then the culture medium was replaced with fresh medium containing the appropriate concentration of the selected glucose metabolism inhibitors. Glucose uptake inhibitors phloretin (PHL) in 10, 25, 50, and 100 µM concentrations; quercetin (Que) and quercetin-3′-sulfate (Q3′S) in 10, 25, 50, and 75 µM concentrations; cytochalasin B in 0.1, 0.5, 1, and 5 µM concentrations; and anti-GLUT1 antibody in 1, 2, 4, and 8 µg/mL concentrations were used. Glycolytic enzyme inhibitors sodium fluoride (NaF) in 2.5, 5, 10, and 20 mM and 3-bromopyruvate (3-BP) in 5, 10, 20, and 50 µM concentrations were tested. Inhibitors of terminal oxidation sodium azide (NaN_3_) in 2.5, 5, 10, and 20 mM and oligomycin A in 25, 50, 75, and 100 µM concentrations were applied. Complex effects of ochratoxin A (OTA) were investigated in 5, 10, 20, and 50 µM concentrations. All treated cells were incubated for 4 h in DMEM before analysis. For the microplate assay, each treatment was repeated in 6 independent plates with 8 technical replicates/treatment/plate. The flow cytometry measurements were performed in triplicate.

### 4.3. Glucose/ATP/Protein Extraction and Measuring Reagents

We developed a glucose reagent (GR) for simultaneous extraction and measurement of glucose, ATP, and cellular protein. First, glucose extraction (GEX) solution was prepared (0.1 M Tris/acetate, 10 mM EDTA-Na_2_, 0.1% TX-100, 5 mM NaF). GEX supplemented with 16 µM Ampliflu, 4.5 µU/mL GOD, and 0.13 µU/mL POD provided the final GR solution. Both GEX and GR were freshly prepared and kept on ice before the measurements, protected from light. ATP was quantified from the GEX-extracted cell lysates after 5 min extraction time, while total cellular protein was measured after glucose determination of the samples.

### 4.4. Validation of Glucose Assay

Our modified glucose assay was based on the principles of the Amplex Red Glucose Oxidase Assay Kit (Invitrogen, Carlsbad, CA, USA), where resorufin, a red fluorescent molecule, is produced in equimolar amount to glucose. In all experiments, in-house glucose standards (PhHg Eur 8) or commercial products (Roche, Mannheim, Germany) were dissolved in GEX without Ampliflu and the enzymes. For cell-free measurements, 100 µL glucose standards (0–10 µM) were pipetted into 96-well plates, then 100 µL of GR with enzymes and Ampliflu was added. After 30 min of incubation at 25 °C in the dark, fluorescence intensity was measured at 540 nm excitation/580 nm emission wavelengths using a Perkin Elmer Enspire multimode reader. The calibration curve was obtained after logistic fitting by using the OriginLab Pro 2016 program.

When dealing with cell cultures, the plates were washed thoroughly but gently 3 times with magnesium and calcium containing PBS by inverting and tapping the plates onto tissue paper, then cells were extracted with 100 µL GEX for 5 min. After extraction, 100 µL of GR with enzymes and Ampliflu was pipetted into each well and fluorescence was read as described above.

The validation protocol of our glucose assay was based on the second edition of Eurachem guidelines [57]. The limit of blank (LOB) was calculated from 88 independent blank samples, while the limit of detection (LOD) and limit of quantification (LOQ) were determined based on 72 analyses [58].

For intra- and interday (interassay) imprecision measurements in cell-free environment, we used 0.5, 1.0, 2.5 and 5.0 µM glucose concentrations, respectively. For intra-assay analysis, 32 parallel measurements were performed on the same day, while interassay imprecision was calculated from data measured on 5 consecutive days with independent calibrations (*n* = 5 × 32 replicates).

Using HepG2 cell cultures, for intra- and interassay imprecision and recovery measurements, we spiked the cells with glucose in the GEX extraction reagent (0.5 µM and 2.5 µM glucose, respectively). The incubation and measurement were carried out as described above. Five independent experiments were performed with 32 technical replicates (*n* = 5 × 32).

Linearity was analyzed by 3 parallel measurements and 8 technical replicates of 5 dilutions of the GEX cell lysates in the range of 20–100% of the original glucose concentrations.

To assess glucose stability in the lysates, glucose determination was performed every consecutive 10 min up to 60 min from the same GEX cell lysates (8 replicates for each time point).

### 4.5. Intracellular Glucose, ATP, and Protein Assay

After various treatments, the adherent cell cultures were washed with PBS containing calcium and magnesium, then extraction was performed by 100 µL GEX extraction solution without enzymes and Ampliflu. After 5 min, 10 µL of cell extract was pipetted into white 96-well optical plates containing 100 µL of bioluminescence ATP reagent, and luminescence signal was measured immediately in the multimode reader [16]. ATP standards were prepared in a separate plate in the range of 17–546 nM ATP. Immediately after sampling for ATP, 100 µL/well GR with enzymes and Ampliflu was added to each well and fluorescence intensities were quantified (30 min incubation at 25 °C, protected from light, *λ*exc = 540 nm, *λ*em = 580 nm wavelengths). Intracellular glucose concentrations were calculated from the equation obtained for the glucose standards as described above in the validation paragraph.

To determine total cellular protein levels, we carried out the Bradford method. BSA dissolved in GEX solution in the range of 20–100 mg/mL served as standard. For the measurement, 20 μL lysate/standard was mixed with 200 μL Bradford reagent. Absorbance was read at 595 nm on the plate reader [16,55].

All results are expressed as mean in percentage compared with the data obtained for the nontreated controls (~100%), and error bars indicate standard deviation (SD).

### 4.6. Measurement of Lactate Production

Lactate from the culture medium of treated cells was measured using a standard laboratory method (plasma lactate kit and Cobas Integra plus 400 analyzer, both from Roche). The effects of the treatments on lactate production were tested after exposure of the lowest and highest concentration of all studied compounds (3 independent parallel measurements on different days).

### 4.7. 2-NBDG Uptake by Flow Cytometry

In previous experiments, we defined the optimal concentration and incubation time of 2-NBDG staining on HepG2 cells to be 60 µM and 1 h. Following treatments with the aforementioned compounds carried out in 6-well plates, the medium was discarded and cells were washed twice with Ca-Mg-PBS. 2-NBDG was added to the wells at a working concentration of 60 µM each, and plates were kept in a humidified incubator containing 5% CO_2_. Uptake of the fluorescent analog was stopped by discarding the supernatant after 1 h and washing the cells 2 times with PBS. Cells were then trypsinized and transferred into flow cytometry tubes (BD Falcon, BD Biosciences, Bedford, MA, USA). Following centrifugation at 400 *g* for 5 min at 4 °C, trypsin was discarded, and cells were washed and centrifuged in PBS once. PI was added at a concentration of 1 µg/mL. After 15 min incubation, cells were centrifuged and resuspended in 300 µL PBS. All tubes were kept on ice to slow down their metabolism and avoid decomposition of the 2-NBDG. Flow cytometry measurements were performed immediately on a BD FACS Canto II instrument (Becton Dickinson and Company). In our gating strategy, first we applied a time gate to check flow stability, doublets were discriminated by forward scatter height and area (FSC-H/FSC-A), and cells were identified by side scatter and forward scatter area (SSC-A/FSC-A). Using PI, we determined the effect of the treatments on cell death, and the fluorescence intensity of 2-NBDG was measured in the fluorescein isothiocyanate (FITC) channel. Despite having only 2 fluorochromes to detect, fluorescence minus one (FMO) controls were applied to set the gates properly. Data were also calculated for the ungated populations (live and dead cells together). Compensation and analysis were carried out with FlowJo software, version 10 (FlowJo LLC, Ashland, OR, USA).

### 4.8. Statistical Evaluation

Microplate treatments were applied on 6 independent plates with 8 replicates/plate/treatment. Data from each plate and each treatment were averaged, SD calculated, and expressed as percentage of the untreated control mean ± SD (~100% ± SD). One-way ANOVA test was used with IBM SPSS Statistics software (version 23), where treatment data were compared to control data. The level of significance was set at *p* < 0.05.

Flow cytometry experiments were measured in triplicate, and we collected 50,000 events from each tube. The measured data did not show normal distribution, thus the median values of 2-NBDG intensity were exported from the flow cytometry analysis software. The exported data showed normal distribution, thus fluorescence intensity of 2-NBDG in the statistical analysis is interpreted as a mean of medians with standard deviation. Unpaired t-test was performed to reveal between-group differences. Level of significance was set at *p* < 0.05. Statistical analysis was carried out using Prism 5 for Windows (GraphPad Software, San Diego, CA, USA).

## 5. Conclusions

We developed and validated an enzymatic fluorescence intracellular microplate glucose method using glucose oxidase/peroxidase enzymes and Ampliflu as a reporter molecule. The HepG2 liver cancer cell line was used for the validation process and for different treatments affecting glucose uptake and/or metabolism. We introduced a nonionic detergent-based lysis buffer that stabilizes glucose, ATP, and cellular proteins at the same time. The analytical performance of our novel method was superior to commercially available methods. In order to support our glucose data, flow cytometry assay was also introduced to monitor cellular glucose uptake using 2-NBDG fluorescent glucose analog. The microplate method showed dose-dependent effects of 10 different compounds in HepG2 cells regarding their glucose and ATP contents. By flow cytometry, we measured glucose uptake both for live cells only and for all cells (without excluding the dead ones). Discrimination between live and dead populations was not possible in the microplate experiments. Glucose content and glucose uptake of the cells was not always identical, which might be explained by the extra 1 h incubation of the cells with the glucose analog without the treating compounds. However, taken together, the results obtained by the two different approaches provide information on the mode of action of the treating agents. The microplate method can also be considered as a multiparametric viability assay to obtain viability data on a large-scale basis with an analysis of time/plate less than one hour.

## Figures and Tables

**Table 1:** Intra- and interassay imprecision and recovery of the glucose assay in aqueous environment using different standards/controls.

	Intra-Assay (*n* = 32)			Interassay (*n* = 160)		
Glucose (µM)	Mean ± SD (µM)	CV (%)	Recovery (%)	Mean ± SD (µM)	CV (%)	Recovery (%)
0.5 ^a^	0.47 ± 0.02	4.26	94.06	0.47 ± 0.04	8.51	95.04
2.5 ^a^	2.55 ± 0.11	4.31	102.09	2.52 ± 0.13	5.16	100.80
1.0 ^b^	0.99 ± 0.04	4.04	99.00	0.97 ± 0.08	8.24	97.00
5.0 ^b^	4.59 ± 0.12	2.61	91.80	4.49 ± 0.25	5.56	89.80

^a^ Standardized by homemade calibrators and controls. ^b^ Standardized by commercial calibrator and diluted from commercial serum-based glucose control (Cfas and PreciControl Universal, both from Roche, Mannheim, Germany; target values given for UV-hexokinase/glucose-6P-dehydrogenase method). SD, standard deviation; CV, coefficient of variation.

**Table 2:** Intra- and interassay imprecision and recovery of the glucose assay in HepG2 cell cultures.

	Intra-Assay (*n* = 32)				Interassay (*n* = 160)			
	Mean ± SD (µM)	CV (%)	Recovery (µM)	Recovery (%)	Mean ± SD (µM)	CV (%)	Recovery (µM)	Recovery (%)
HepG2 cells	0.95 ± 0.07	8.06	n.a.	n.a.	0.94 ± 0.11	12.11	n.a.	n.a.
HepG2 + 0.5 µM glucose	1.38 ± 0.13	9.45	0.43	91.81	1.37 ± 0.15	11.03	0.43	90.41
HepG2 + 2.5 µM glucose	3.00 ± 0.21	7.25	2.06	83.70	3.03 ± 0.24	8.03	2.10	83.70

SD, standard deviation; CV, coefficient of variation; n.a., not applicable.

**Table 3:** Extracellular lactate levels in culture medium after various treatments of HepG2 cells (T = 4 h).

Mode of Action	Treatment	Lactate ± SD (%)
DMEM	-	57 ± 7
Control cells in DMEM	-	100 ± 29
Inhibitors of glucose transporters	10 µM PHL	78 ± 19
	100 µM PHL	98 ± 20
	10 µM Que	104 ± 30
	75 µM Que	91 ± 24
	10 µM Q3′S	105 ± 30
	75 µM Q3′S	100 ± 28
Inhibitors of glycolysis	2.5 mM NaF	65 ± 12
	20 mM NaF	27 ± 8
	5 µM 3-BP	76 ± 19
	50 µM 3-BP	71 ± 18
Inhibitors of terminal oxidation	2.5 mM NaN_3_	114 ± 28
	20 mM NaN_3_	138 ± 33
	25 µM oligomycin A	113 ± 23
	100 µM oligomycin A	93 ± 29
Complex effect	5 µM OTA	106 ± 27
	50 µM OTA	95 ± 30

Data are expressed as % of control values (Dulbecco’s Modified Eagle’s Medium (DMEM) with untreated cells, mean ± SD) from three independent experiments. PHL, phloretin; Que, quercetin; Q3′S, quercetin-3′-sulfate; OTA, ochratoxin A; 3-BP, 3-bromopyruvate.

## Supplementary Materials

Supplementary materials can be found at http://www.mdpi.com/1422-0067/19/9/2670/s1.
