# Supplementary material for: A One-Step Extraction and Luminescence Assay for Quantifying Glucose and ATP Levels in Cultured HepG2 Cells
Source: Int J Mol Sci. 2018 Sep 8;19(9):2670. doi: 10.3390/ijms19092670 (PMC6163413; doi:10.3390/ijms19092670)
Supplement: Supplementary file 1 [file ijms-19-02670-s001.pdf]

International Journal of *Molecular Sciences*

MDPI

Supplementary Material

A One-Step Extraction and Luminescence Assay for Quantifying Glucose and ATP Levels in Cultured HepG2 Cells

Rita Csepregi [1,3], Viktória Temesfői [1,3], Nikolett Sali [1], Miklós Poór [2,3], Paul W. Needs [4], Paul A. Kroon [4], and Tamás Kőszegi [1,3,*]

1 Department of Laboratory Medicine, University of Pécs, Medical School, H-7624 Pécs, Ifjúság u. 13, Hungary; ritacsepregi93@gmail.com (R.C.); vtemesfoi@gmail.com (V.T.); niki26@gmail.hu (N.S.)

2 Department of Pharmacology, University of Pécs, Faculty of Pharmacy, H-7624 Pécs, Szigeti u. 12, Hungary; poor.miklos@pte.hu (M.P.)

3 János Szentágothai Research Center, H-7624 Pécs, Ifjúság u. 20, Hungary

4 Quadram Institute Bioscience, Norwich Research Park, Norwich NR4 7UA, UK; paul.needs@quadram.ac.uk (P.N.); paul.kroon@quadram.ac.uk (P.K.)

* Correspondence: tamas.koszegi@aok.pte.hu; Tel.: +36-30-491-7719; Fax: +36-72-536-121

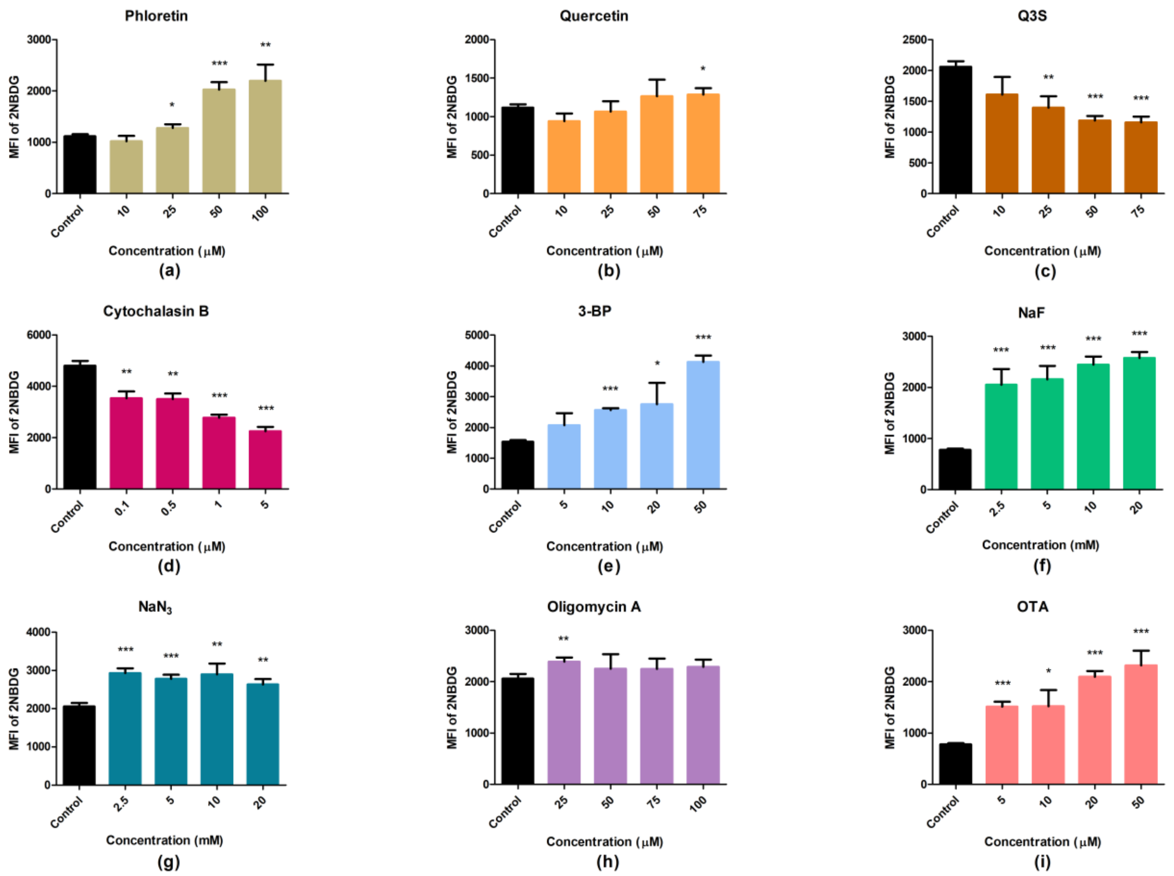

Supplementary Figure 1. 2-NBDG uptake in HepG2 cells as results of (**a**) phloretin, (**b**) quercetin, (**c**) Q3'S, (**d**) cytochalasin B, (**e**) 3-BP, (**f**) NaF, (**g**) NaN_3, (**h**) oligomycin A, and (**i**) OTA treatments. Incubation time with metabolic inhibitors: 4 h, 2-NBDG: 1 h, MFI: mean fluorescence intensity. Columns represent the mean of medians, error bars show the interquartile range of fluorescence intensity of intracellular 2-NBDG. PI positive cells are included into the analysis. (* p <0.05, ** p < 0.01 *** p < 0.001 compared with the controls).
